# Pregnancy Changes the Response of the Vomeronasal and Olfactory Systems to Pups in Mice

**DOI:** 10.3389/fncel.2020.593309

**Published:** 2020-12-18

**Authors:** Cinta Navarro-Moreno, Maria Jose Sanchez-Catalan, Manuela Barneo-Muñoz, Rafael Goterris-Cerisuelo, Maria Belles, Enrique Lanuza, Carmen Agustin-Pavon, Fernando Martinez-Garcia

**Affiliations:** ^1^Lab of Functional Neuroanatomy (NeuroFun-UJI-UV), Unitat Predepartamental de Medicina, Faculty of Health Sciences, Universitat Jaume I, Castellón de la Plana, Spain; ^2^Lab of Functional Neuroanatomy (NeuroFun-UJI-UV), Departament de Biologia Cellular, Funcional i Antropologia, Faculty of Biological Sciences, Universitat de València, Valencia, Spain

**Keywords:** pregnancy, pup chemosignals, vomeronasal system, olfactory system, mice, IEGs

## Abstract

Motherhood entails changes in behavior with increased motivation for pups, induced in part by pregnancy hormones acting upon the brain. This work explores whether this alters sensory processing of pup-derived chemosignals. To do so, we analyse the expression of immediate early genes (IEGs) in the vomeronasal organ (VNO; Egr1) and centers of the olfactory and vomeronasal brain pathways (cFos) in virgin and late-pregnant females exposed to pups, as compared to buttons (socially neutral control). In pup-exposed females, we quantified diverse behaviors including pup retrieval, sniffing, pup-directed attack, nest building and time in nest or on nest, as well as time off nest. Pups induce Egr1 expression in the VNO of females, irrespective of their physiological condition, thus suggesting the existence of VNO-detected pup chemosignals. A similar situation is found in the accessory olfactory bulb (AOB) and posteromedial part of the medial bed nucleus of the stria terminalis (BSTMPM). By contrast, in the medial amygdala and posteromedial cortical amygdala (PMCo), responses to pups-vs-buttons are different in virgin and late-pregnant females, thus suggesting altered sensory processing during late pregnancy. The olfactory system also shows changes in sensory processing with pregnancy. In the main olfactory bulbs, as well as the anterior and posterior piriform cortex, buttons activate cFos expression in virgins more than in pregnant females. By contrast, in the anterior and especially posterior piriform cortex, pregnant females show more activation by pups than buttons. Correlation between IEGs expression and behavior suggests the existence of two vomeronasal subsystems: one associated to pup care (with PMCo as its main center) and another related to pup-directed aggression observed in some pregnant females (with the BSTMPM as the main nucleus). Our data also suggest a coactivation of the olfactory and vomeronasal systems during interaction with pups in pregnant females.

## Introduction

Maternal behavior can be defined as any interaction of an adult female with infant conspecifics that helps the latter to survive until their maturity (Numan and Insel, 2003). Maternal behavior has therefore a strong impact on reproductive success, but it is also very beneficial for infant neurodevelopment (Curley and Champagne, 2016). Indeed, well-adapted mammalian dams are frequently engaged in devoted maternal care (pup-directed behaviors) consisting of retrieving the pups to the nest, crouching over the pups to keep them warm and nurturing them by means of lactation while frequently licking-grooming their bodies. In addition, dams also show an intense activity not directed to pups, such as building and maintaining the nest already before parturition and defending it against adult conspecific intruders that might constitute a threat for their pups (maternal aggression) (Numan and Insel, 2003).

The enormous investment of time and energy that these behaviors require may explain why females only exhibit fully motivated maternal behavior during peripartum (for a review see Kohl et al., 2017; Salais-López et al., 2020). Although maternal behaviors are normally expressed after delivery, when pups are present, they are already facilitated during pregnancy. Thus, pregnant females already show nest building (Lisk, 1971) and maternal aggression (prepartum aggression, Mann et al., 1984). In addition, it was shown that primigravid female rats that were hysterectomized before parturition, also displayed facilitated pup-directed behaviors (Rosenblatt and Siegel, 1975; Bridges et al., 1978). By contrast, it has been reported that pregnancy also facilitates pup attacks in both rats (Peters and Kristal, 1983; Mayer and Rosenblatt, 1984) and mice (McCarthy and Vom Saal, 1985). Although this may seem a contradiction in terms, infanticide may constitute an adaptive behavior during motherhood in some circumstances (Blaffer Hrdy, 1979; Latham and Mason, 2004; Kuroda and Tsuneoka, 2013) and this may include late pregnancy.

The most likely mechanism underlying this timely, temporary enhancement of maternal responses to pups is their facilitation by hormones associated to pregnancy, as indeed it has been demonstrated for several mammalian species (Bridges, 2020). For instance, sexual steroids together with prolactin and/or placental lactogens (Bridges and Ronsheim, 1990; Bridges and Freemark, 1995), acting onto centers of the sociosexual brain network (singularly the medial preoptic area; Brown et al., 2017), accelerate the onset of maternal behaviors in virgin rats.

Therefore, the current view of the neurobiology of motherhood assumes that hormonal events of late pregnancy prime specific brain circuits mediating maternal behaviors (the socio-sexual brain network), so that parturient females react properly to infant-derived stimuli, but once maternal behavior is initiated, it continues without the need of further hormonal regulation (Numan and Insel, 2003).

In this context, it is important to understand what sensory channels are involved in the detection of the relevant pup stimuli. Although the identity of specific pup chemosignals has not been elucidated yet, since rodents are macrosmatic animals it is likely that pup-derived chemosignals have a critical role in eliciting maternal behavior. In fact, altered chemosensing has dramatic consequences on maternal responses in rodents. Thus, bulbectomy (Gandelman et al., 1971; Vandenbergh, 1973) and nasal epithelium lesions (Seegal and Denenberg, 1974), result in nearly systematic pup-killing by lactating females. Moreover, null mutations of genes encoding critical molecules for olfactory transduction not only result in anosmia, but also lead to maternal neglect of pups and deficient nest maintenance (Belluscio et al., 1998; Wang and Storm, 2011). By contrast, null-trpc2 mice, whose vomeronasal organ (VNO) is not functional (Leypold et al., 2002; Stowers et al., 2002), show just reduced maternal care (Kimchi et al., 2007), as well as deficient nest maintenance but complete lack of maternal aggression (Leypold et al., 2002; Hasen and Gammie, 2011). In addition, Lepri et al. (1985) reported reduced pup retrieval after VNO ablation. Together, these findings suggest a key role of chemosensory olfactory stimuli in maternal care. By contrast, vomeronasal stimuli seem to play a clear role in aggression, including maternal nest defense, but there is conflicting evidence on its function in pup-directed maternal behaviors.

Conversely, the VNO is critical for the response of males to pups. First, VNO ablation reduces infanticide in sexually naïve males (Tachikawa et al., 2013). Also, targeted mutations abolishing VNO function (trpc2 knockout, Nakahara et al., 2016) provoke paternal behavior, inducing pup care similar to that of lactating dams. Surprisingly, the VNO of mice possess a population of cells that express non-canonically a receptor of the olfactory family (olf692) that has been related to pup-derived odor detection. In sex-naïve males, which are infanticidal, a high proportion of olf692-expressing VNO cells are activated by pup odors. In contrast, in paternal males and in females (irrespective of their status, virgins or dams) a much smaller proportion of these cells are activated following pup exposure (Nakahara et al., 2016).

All these data indicate that pup chemosignals are important in the response of adult rodents to infants, and in females this is especially critical during motherhood, when altered chemosensing has a strong impact on maternal behaviors. However, the specific role of each sensory channel, e.g., olfactory and vomeronasal, in this communication is still unclear. In addition, there is a surprising lack of information on possible functional changes in these systems induced by pregnancy hormones, which might explain, at least in part, the enhanced reinforcing properties of pups for females during motherhood (Hauser and Gandelman, 1985; Salais-López et al., 2017, 2020).

Thus, to study the possible changes in both main and accessory olfactory systems during pregnancy, we recorded and scored the behavior of late-pregnant (LP) (E18) and virgin female mice in response to pups' exposure. Afterwards, in these females, we assessed activation of the VNO by means of immunohistochemical detection of Egr1 expression, and the primary and secondary olfactory and vomeronasal brain centers by means of cFos detection. As a control stimulus, we used a non-social object (buttons) of approximately the same size as pups. Since both variables (behavior and brain activation) were measured in the same animals, we were able to analyse possible correlations between brain activity and specific aspects of maternal behavior. The results confirm the presence of pup-derived chemosignals activating the VNO of females and suggest changes in stimulus processing in both chemosensory systems during late pregnancy. In addition, these findings suggest the existence of two distinct pathways in the vomeronasal system of females related to pup care and pup-directed attacks, respectively.

## Materials and Methods

### Animals

For the present study, we used 10-weeks-old virgin female mice (*n* = 16) and late-pregnant female mice (*n* = 14) of the CD1 strain. Late-pregnant mice (LP) were bred in the animal facility and parturition (usually occurring at gestational day 19) was expected 1–2 days after behavioral testing. Females were housed in homologous pairs, in order to avoid isolation stress. Pairs of same condition females were housed together at least 20 days before the experiment (pairs of LP females were mated by the same male) in polypropylene cages with a controlled temperature of ~24°C and a 12-hr light/dark cycle (lights on at 08:00 h) with *ad libitum* water and food supply. The pregnant day was considered as the one in which a pair of LP females were mated with a male (housed together overnight). Experimental procedures were approved by the Committee of Ethics and Animal Experimentation of the Universitat Jaume I and treated throughout according to the European Union Council Directive of June 3rd, 2010 (6106/1/10 REV1).

### Experimental Design and Behavior Analysis

Experimental females were exposed to pups or buttons, plastic and round objects from similar size than pups, which constitute socially neutral stimuli. Pups in postnatal day 4 were obtained from different female donors. Thus, we used four female groups: (1) LP exposed to pups, (2) virgins exposed to pups, (3) LP exposed to buttons, and (4) virgins exposed to buttons.

Two days prior the behavioral testing, females underwent a habituation phase. Eight glass marbles were deposited in the females' home cage once per day for 2 days at the time in which experiments were scheduled to be performed, in order to habituate the animals to the procedure. In the test day, pairs of virgins and of pregnant female mice were exposed to eight buttons or eight pups, placed in distal areas of the home cage relative to the nest (consisting on pieces of shredded paper). Buttons (**Figure 2A**) were round, white, with four holes and made of plastic. Two different sizes (13 and 20 mm in diameter) but similar weight (0.69 and 0.63 g, respectively) were used. Four buttons of each class were introduced in each cage. The behavior of the females exposed to pups was video recorded for 90 min, although observation of maternal behavior was restricted to the first 8 min since it is mainly expressed immediately following pup introduction (Martín-Sánchez et al., 2015) and may better reflect the expression of IEGs observed, which reaches its maximum 60–90 min after stimulation occurred (Hoffman et al., 1993). Within these 8 min, 32 5-s periods were analyzed (four 5-s periods per min, separated by 10-s intervals). For each 5-s period, we registered the most maternal behavior exhibited by the female, according to the following hierarchy: *pup retrieval*, females carried the pups to the nest; *in nest*, females stayed inside the nest in close contact with pups; *nest building*, females gathered pieces of nest material; *on nest*, females were located on the nest, near the pups but not in contact with them; *approach to pups*, olfactory exploration of pups out of the nest, not followed by retrieval; and *off nest*, females were out of the nest and show no interaction with pups. Then, 32 behavioral events were registered in each animal, distributed among the items described above. Nests were big and well-organized so that during in-nest periods the female and the pups could not be observed. Therefore, specific pup-care items occurring within the nest (licking grooming, arch-back posture of the female) were not assessed.

Moreover, those behavioral items were used to calculate maternal and chemosensory scores for each animal. The maternal score is a weighted sum of those episodes in which female's behavior reflects a maternal state (pup retrieval, nest building, in nest, and on nest):

$$\begin{array}{l} Maternal\;Score=5\;x\;Retrieval+5\;x\;In\;Nest\\ \;+4\;x\;Nest\;Building+2\;x\;On\;Nest \end{array}$$

In the same way, the chemosensory score is composed of a weighted sum of episodes in which the females are likely interacting and sniffing at pups:

$$\begin{array}{l} Chemosensory\;Score=5\;x\;In\;Nest+3\;x\;Retrieval\\ \;+3\;x\;Approach\;To\;Pups+1\;x\;On\;Nest \end{array}$$

At the end of experiment, we observed 1–3 pups killed, sometimes partially mutilated, in the cages of LP females. Then, we revised the video/audio-recordings and identified those moments in which pup-directed attacks occurred, which were easy to recognize as they always occurred while the female was out of the nest, licking-grooming a pup, which suddenly started emitting strong distress vocalizations which stopped after a few seconds. We measured the latency to each attack to a pup and assigned it to the female that displayed pup-directed aggression. For each female we calculated a pup aggression score:

$$\begin{array}{l} Pup\;Aggression\;Score=\overset{i=8}{\underset{i=1}{\sum }}\left(25-latency\;to\;attack\;pup\;i\right) \end{array}$$

A latency of 25 min was assigned for those females not attacking pups (all pup attacks occurred during the first 24 min). This way, pup aggression score was zero for the females not expressing any pup-directed aggression, and it was higher for those females attacking more pups and/or attacking pups with a lower latency.

Finally, the interaction between females in the same cage was also measured for each of these 32 5-s periods as present (1) or not present (0), considering an interaction when a female sniffed the other.

Since we were initially interested only in pup-directed behaviors, we did not record behavioral responses of the females exposed to buttons.

### Tissue Processing and Immunohistochemistry

Following 90 min of stimulus introduction, females were overdosed with an intraperitoneal injection of sodium pentobarbital (Vetoquinol, Madrid, Spain; 0.02 mg/g of body weight, Shipley and Adamek, 1984) and transcardially perfused with 4% paraformaldehyde (PFA) in 0.1 M phosphate buffer (PB), pH 7.4. Brains were dissected from the skull, snouts were separated from the skull and muscles removed in order to obtain a block with the VNO. Both, brains and snouts were post-fixed overnight in 4% PFA at 4°C. After fixation, snouts were washed 3 × 10′ in 0.01 M phosphate buffer (PB) with 0.9% NaCl (PBS), decalcified using 250 mM EDTA in 0.1 M PB during 5 days at 4°C and washed 3 × 10′ in 0.05 M Tris Buffer (TB) with 0.9% NaCl (TBS), pH 7.6. Then they were placed into a cast of warm 15% gelatine in 0.05 M TB, kept at 4°C overnight, trimmed and placed in 4% formaldehyde in 0.1 M PB for 2 h at 4°C.

Brains and snout blocks were cryoprotected in 30% sucrose in 0.01 M PB at 4°C until they sank, and then coronal sections (snouts 30 μm-thick; brains 40 μm-thick) were obtained using a freezing microtome (Microm HM-450, Walldorf, Germany), collected in five parallel series in 30% sucrose in PB and stored at −20°C.

One series of snout sections of each animal was processed for free-floating immunohistochemistry of Egr-1 protein, in order to assess the activity of VNO sensory neurons (Isogai et al., 2011). To do so, sections were (a) rinsed 4 × 5 min in TBS; (b) immersed in 1% H_2_O_2_ and 0,3% Triton X-100 in TBS solution for 30 min for endogenous peroxidase inhibition; (c) rinsed 3 × 5 min in TBS; (d) immersed for an hour in a blocking solution containing 4% normal goat serum and 0.3% Triton X-100 in TBS 0.01 M, pH 8; (e) incubated overnight at room temperature with the primary antibody (rabbit anti-Egr1, no. 4153S; Cell Signaling Technology) diluted 1:500 in the blocking solution; (f) rinsed 5 × 5 min in TBS; (g) incubated in 1:400 dilution of biotinylated goat anti-rabbit secondary antibody (Vector BA1000) in the blocking solution for 2 h; (h) rinsed 5 × 5 min in TBS; (i) transferred to 1:50 avidin-biotin-peroxidase complex (Vectastain-Elite, Vector Laboratories) in TBS for 90 min; (j) rinsed 3 × 5 min in TBS and 3 × 5 min in 0.05 M TB, pH 7.6; and finally, (k) the peroxidase activity was revealed with diaminobenzidine tetrahydrochloride (DAB) reaction (0.025% DAB and 0.01% H_2_O_2_ in TB). The reaction was stopped by successive rinsing of sections in TB. Sections were mounted on slides and coverslipped in DPX (Scharlau Laboratory).

In parallel, a series of brain free-floating sections were processed for cFos immunohistochemistry. Sections were (a) rinsed 3 × 10 min in TBS; (b) immersed in 1% H_2_O_2_ in TBS solution for 30 min for endogenous peroxidase inhibition; (c) rinsed 3 × 10 min in TBS; (d) immersed for an hour in a blocking solution containing 3% normal goat serum, 3% bovine serum and 0.3% Triton X-100 in 0.01 M TBS, pH 8; (e) incubated overnight at room temperature with the primary antibody (rabbit anti-cFos n°. 226003; Synaptic Systems) diluted 1:5,000 in the blocking solution; (f) rinsed 3 × 10 min in TBS; (g) incubated in 1:200 dilution of biotinylated goat anti-rabbit secondary antibody (Vector BA1000) in the blocking solution for 2 h; (h) rinsed 3 × 10 min in TBS; (i) transferred to 1:50 avidin-biotin-peroxidase complex (Vectastain-Elite, Vector Laboratories) in TBS for 90 min; (j) rinsed 2 × 10 min in TBS and 2 × 10 min in TB (Tris Buffer 0.05 M pH 7.6); and finally, (k) the peroxidase activity was revealed with diaminobenzidine tetrahydrochloride (DAB) reaction (0.025% DAB and 0.01% H_2_O_2_ in TB). The reaction was stopped by successive rinsing of sections in TB. Sections were mounted on slides and coverslipped in DPX (Scharlau Laboratory).

For each immunohistochemistry (Egr1 and cFos), sections of animals of the different groups (LP and virgin females exposed to buttons and pups) were processed simultaneously using the same batches of reagents and antibodies, in order to minimize inter-individual variability and to avoid inter-group bias.

### Image Analysis

For the assessment of Egr1 expression, images of all VNO sections of a series (1 in 5) were acquired at 10× using a digital camera (DFC495) attached to a microscope Leitz DMRG (Leica, AG, Germany) and evaluated with ImageJ (NIH). Acquisition conditions included gamma = 1 and a level of exposure just high enough as to avoid white saturation in void areas of the image. For each picture VNO Egr1 immunoreactive cells (Egr1-ir cells) were manually counted (cell counter tool, ImageJ) by a person who was blind to the experimental conditions of the samples. VNO area was calculated on Image J software (NIH). Then, for each animal, Egr1 density (Egr1-ir cells/mm^2^) was calculated by dividing the total number of Egr1-ir cells counted in all the VNO sections by the total area of these sections.

Expression of cFos was assessed in a selection of brain nuclei involved in chemosensory processing, including nuclei from both vomeronasal and olfactory systems. For the vomeronasal system we sampled the accessory olfactory bulb (AOB, mitral cell layer) and its main synaptic targets, the posteromedial cortical amygdaloid nucleus (PMCo), the medial amygdala (posterodorsal division, MePD) and the medial part of the posteromedial division of the bed nucleus of the stria terminalis (BSTMPM). Concerning the olfactory system, we analyzed the main olfactory bulb (MOB, granular cell layer) and the anterior and posterior divisions of the piriform cortex (PirAnt and PirPost, respectively). The expression of IEGs in the granular layer of the MOB is a good estimator of the activity of the center and reflects the activity of the projection neurons (mitral cells; see Bepari et al., 2012).

For each nucleus, we sampled specific frames at particular anteroposterior levels, as indicated in **Figures 2**, **3** (Paxinos and Franklin, 2004). We acquired images of both hemispheres as described above and, in the case of the MePD, selected a triangle-shaped region of interest to exclude the optic tract (**Figure 2C**). Image processing and analysis were conducted on ImageJ software (NIH). Briefly, the RGB color image was converted to grayscale by selecting the green channel. Images were then binarized setting the threshold at 75% of the mode of the gray histogram, so that every pixel below this threshold was considered labeled. The resulting binary images were further filtered using commands “fill holes,” “open” (3 iterations), and “watershed.” Then, particles were automatically counted, discarding those smaller than half the average size of the cells from that specific nucleus (calculated in turn by measuring the average area of six randomly selected intensely labeled cells in the nucleus).

For most nuclei the density of cFos-ir cells (cells/mm^2^) was calculated by dividing the total number of particles in both hemispheres, by the sum of the areas of the regions of interest. In the AOB and the MOB the high density of cells and intensity of immunostaining made it difficult to separate single cells using the image analysis procedure described above. Therefore, we simply measured the area fraction occupied by labeling after thresholding (immunoreactive area/total area).

### Statistical Analysis

We first compared the behavior of the females (virgins and LP females) exposed to pups. To do so, after testing for normality with the Kolmogorov-Smirnov test, data derived from most of the behavioral events did not follow a normal distribution or showed normality but not homogeneous variance. Then, behavioral differences (behavioral events or scores) between virgin and LP females exposed to pups were evaluated using a two-sample *t*-test for non-homogenous variances (for samples showing normality) or a Wilcoxon test for those displaying no normal distribution.

Regarding the analysis of Egr-1 and c-Fos expression, when data accomplished normality (Kolmogorov-Smirnov test) and homoscedasticity (Levene test), a two-way ANOVA was performed, with “FEMALE” (virgin or LP) and “STIMULUS” (buttons or pups) as factors. Significant FEMALE × STIMULUS interactions were explored by *post-hoc* pairwise comparison with Bonferroni corrections. If data did not fulfill normality and homoscedasticity, we applied the two-way ANOVA after logarithmic transformation (Log10 [n + 1]). If the transformation failed to render normality and/or homoscedasticity, a two-sample *t*-test for non-homogenous variances (for samples showing normality) or a Wilcoxon test for those displaying no normal distribution, was performed with non-transformed data to assess the differences between FEMALE (virgin vs. LP) and between STIMULUS (buttons vs. pups).

When inspecting the VNO sections, we realized that cross-sections through the center of the VNO showed few Egr-1 positive cells whereas, very often, small sections through the tips of the VNO were rich in labeled cells (see **Figures 2B–D**). Therefore, we tested if specific populations located at the ends of the VNO were sensitive to pup-derived stimuli (see Supplementary Figure 1).

After that, we explored Spearman correlations between behavioral data and immediate early gene expression levels (IEGs) (Egr1-ir for the VNO; cFos-ir for the brain) separately in LP and virgin females exposed to pups. This allows investigating the relationship between activity in specific olfactory and vomeronasal nuclei with the expression of specific maternal behaviors and exploring this relationship during pregnancy.

In addition, we also performed Spearman correlation analysis between IEGs expression in the VNO and the different chemosensory brain centers in both groups of pup-exposed females. This allows investigating patterns of neural activity in the centers of the olfactory and vomeronasal systems during interaction with pups and exploring whether hormones acting during late pregnancy may change these patterns of brain activity.

Statistical analysis was performed using SPSS software package (IBM). The significance level was set at *p* < 0.05.

## Results

### Behavior of Late-Pregnant and Virgin Female Mice Following Exposure to Pups

**Figure 1:**
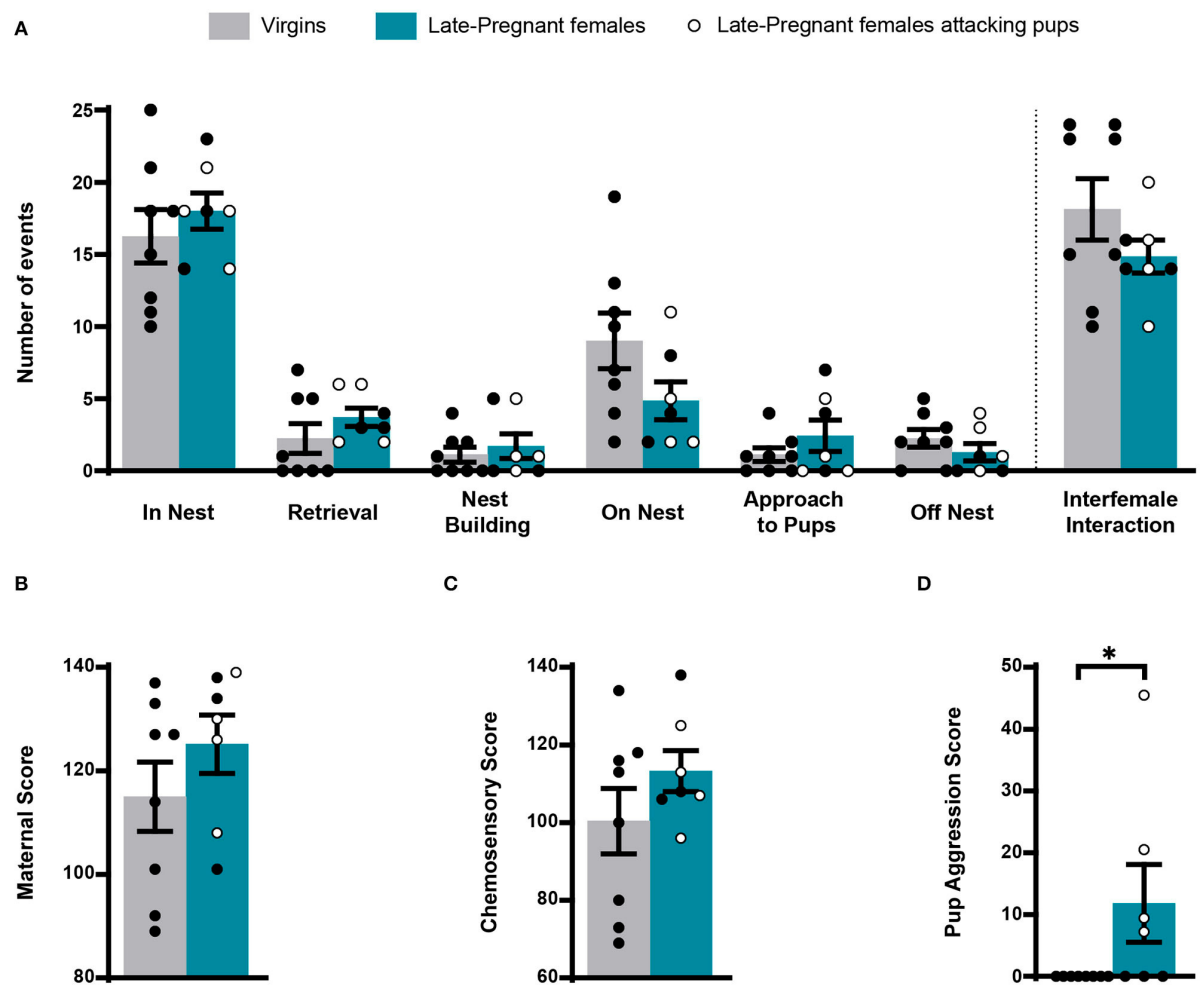
Behavior of virgin and late pregnant (LP) female mice following exposure to pups. Histogram showing the occurrence of maternal behaviors and interfemale interactions (mean ± SEM), scored as number of events during the first 8 min after pup introduction (in nest, retrieval, nest building, on nest, approach to pup and off nest; see text), did not show significant differences between female groups **(A)**. Similarly, the maternal score **(B)** and chemosensory score **(C)** were similar in virgins and late-pregnant females. Finally, the pup aggression score **(D)** was significantly different between female groups, since only some of the LP females performed pup-directed aggression (*p < 0.05). Individual data are also show, with empty circles corresponding to the LP females displaying pup-directed aggression, and black filled ones corresponding to those females not displaying aggression.

During exposure to pups, we scored several of the behavioral events displayed by females: pup retrieval, in nest, nest building, on nest, approach to pups, off nest and pup-directed aggression and interfemale interaction. Statistical analysis revealed non-significant differences for most behaviors between LP and virgin females (pup retrieval, *Z* = −1.289, *p* = 0.197; in nest, *t* = −0.760, *p* = 0.461; nest building, *Z* = −0.368, *p* = 0.713; on nest, *t* = 1.722, *p* = 0.109; approach to pups, *Z* = −0.544, *p* = 0.587; off nest, *t* = 1.105, *p* = 0.289) (Figure 1). In a similar way, there were no differences between females concerning the maternal score (*t* = −1.141, *p* = 0.274) and the chemosensory score (*t* = −1.252, *p* = 0.233). Four out of 7 LP females displayed pup aggression: one attacked three pups, one attacked two pups, and two attacked one pup each. By contrast, virgin females did not attack pups. Accordingly, comparison of pup aggression score rendered significant differences between females (*Z* = −2.376, *p* = 0.017). Finally, interfemale contact did not differ between the LP and virgin females (*Z* = −1.108, *p* = 0.268) (Figure 1).

Overall, these results show that maternal behavior does not differ substantially between LP and virgin females. Also, possible differences in the activity of chemosensory brain centers between females (or the VNO) cannot be attributed to differences in interaction with pups, since with exception of pup-directed aggression, LP and virgin females displayed similar behavior. Moreover, differences in IEGs-ir between females cannot be attributed to interfemale interactions.

### Response of the Vomeronasal System to Pup-Derived Stimuli

One of the aims of this work is to explore the response of the vomeronasal system to possible pup-derived chemosignals detected by the VNO, and the possibility that adult females change their sensitivity to these stimuli and/or their sensory processing mechanisms during late pregnancy. To do so, we analyzed the neuronal response of the VNO and the primary and secondary vomeronasal brain centers by using quantification of IEGs expression in LP and virgin female mice.

**Figure 2:**
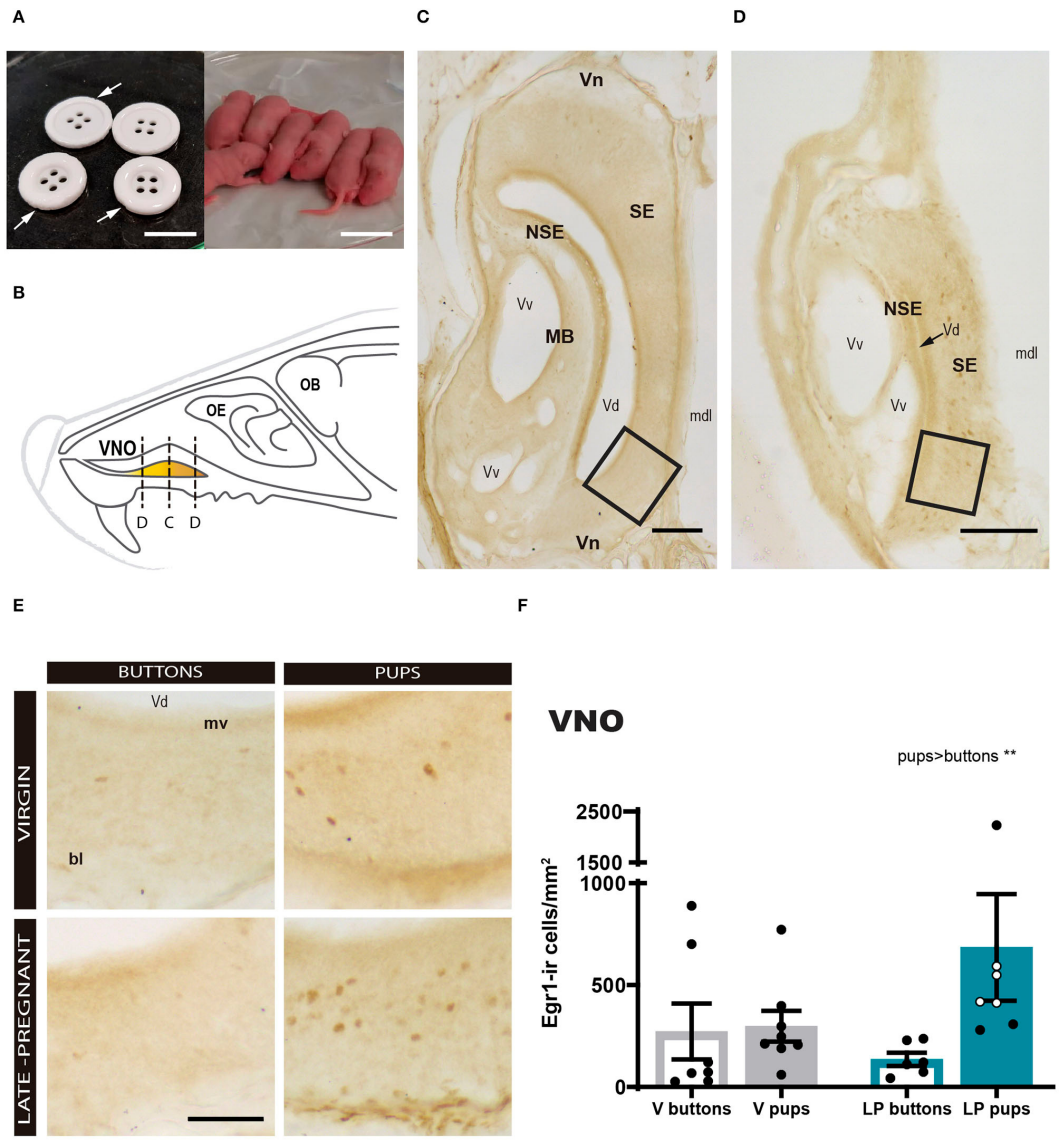
Expression of Egr1 in the vomeronasal organ (VNO) of females (virgin or late-pregnant) exposed to pups or buttons. Buttons are of a size comparable to pups **(A)** and are not avoided but actually gnawed (arrowheads point to notches). Diagram of the VNO **(B)** illustrating the levels of the sections shown in **(C,D)**, which are two low-power photographs of the VNO showing the main anatomical landmarks at two different antero-posterior levels (C, center of the VNO; D, tip of the VNO). Examples similar to the framed areas in **(C,D)** are shown at higher magnification in **(E)** for each experimental group. A bar histogram of raw data (mean ± SEM) of Egr1-positive cells in the VNO of the different groups is shown in **(F)**, where individual data are also plotted. The empty circles correspond to those females displaying pup-directed aggression. Statistical analysis of the density was performed on the log transformed values to achieved normality and homocedasticity (see text). Egr1 expression is increased in response to pups (**p < 0.01) as compared to a socially neutral stimulus (buttons), but there is no difference between virgin and late-pregnant females. Scale bars: **(A)** 2 cm; **(C,D)** 100 μm; **(E)** 50 μm.

First, we analyzed the response of the VNO to pups or buttons exposure in LP and virgin females. A two-way ANOVA of log-transformed Egr1-ir cell density detected a significant main effect for STIMULUS (*F*_1, 24_ = 10.259, *p* = 0.004), but no significant differences for FEMALE (*F*_1, 24_ = 1.249, *p* = 0.275) and no FEMALE × STIMULUS interaction (*F*_1, 24_ = 1.223, *p* = 0.280). As expected, pups induced a higher Egr1-ir cell density in the VNO compared to buttons in both LP and virgin females (Figure 2). This suggests that pups secrete chemosignals that are detected by the VNO of adult females.

We realized that small cross sections, e.g., sections though the tip of the VNO, apparently displayed more Egr1-ir cells than large cross sections, e.g., sections through the center of the organ (compare Figures 2C and D). Therefore, we explored a possible non-homogenous expression of Egr1 in the VNO by performing a correlation analysis between Egr1-ir cell density and section area. The results confirmed that the larger sections display the lower the Egr1-ir cell density (see Supplementary Figure 1). Next, we tested whether this might be due to heterogeneous distribution of specific cell population responding to pups. To do so, we selected the two largest (central) and the two smallest sections (tip) of each animal and analyzed whether they showed different density of Egr1-ir cells in Virgin and LP females exposed to pups and buttons, using a three-way ANOVA (see Figure 1). The results confirmed a strong effect of the stimulus (pups rendered higher density of Egr1-ir cells than buttons; *p* = 0.013) and the level (tip sections having significantly higher density of Egr1-ir cells than central sections; *p* < 0.001). Interactions between factors, LEVEL × FEMALE, LEVEL × STIMULUS, STIMULUS × FEMALE, or LEVEL × STIMULUS × FEMALE were not significant (*p* > 0.6 in all cases). Therefore, although expression of Egr1 was dependent on the stimulus and the level or the VNO (heterogeneous distribution), this was not dependent on the female (does not change with pregnancy) or stimulus.

**Figure 3:**
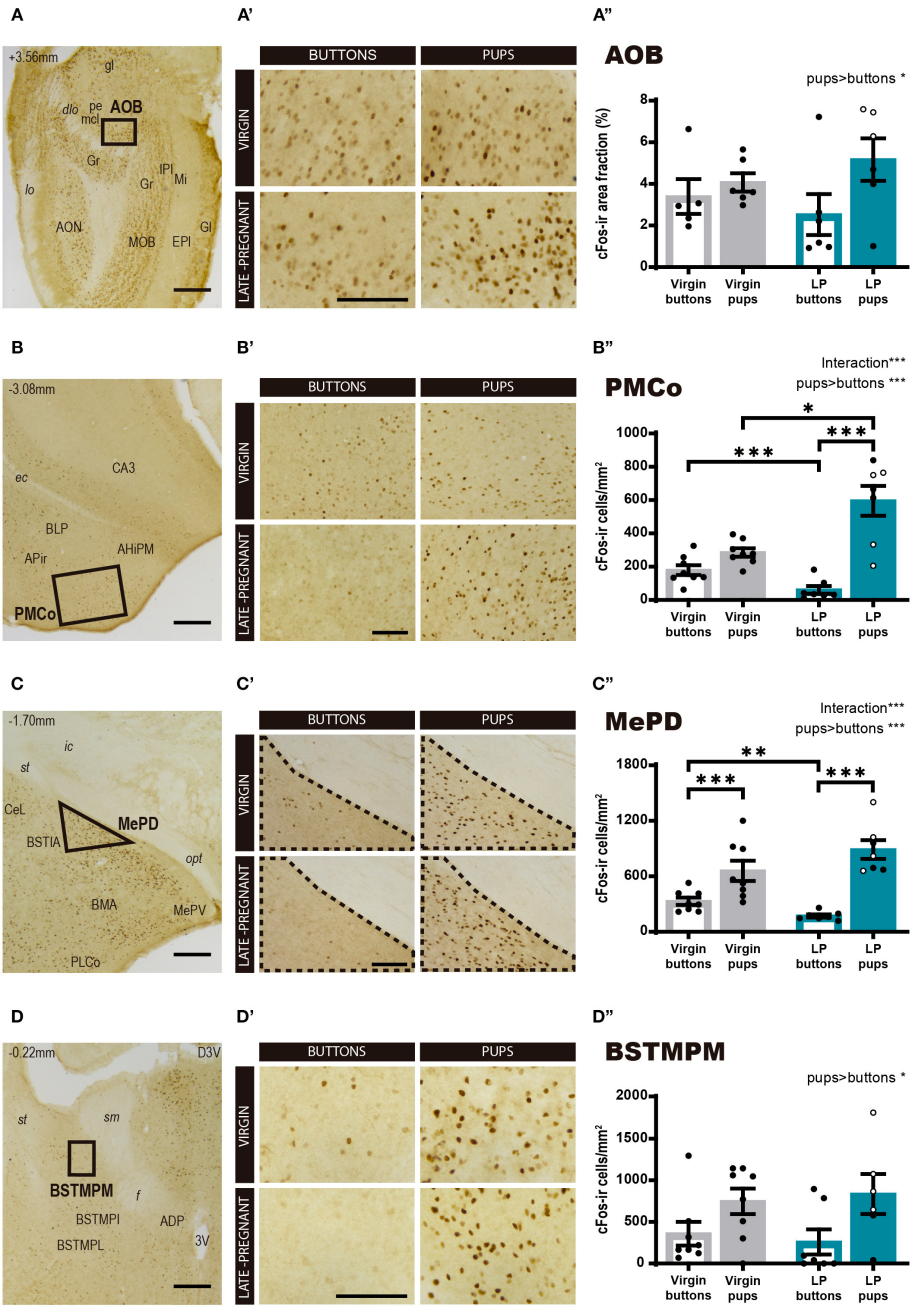
Expression of cFos in brain areas of the vomeronasal system following exposure to pups or non-social control stimulus. **(A–D)** Low power photomicrographs showing cFos expression in the AOB **(A)**, the PMCo **(B)**, the MePD **(C)**, and the BSTMPM **(D)**. The numbers in the left upper part of the images indicate the approximate anteroposterior coordinate of the sections relative to bregma (Paxinos and Franklin, 2004). The framed areas, shown at higher magnification in **(A′-D′)**, indicate the regions where cFos expression was analyzed. **(A′-D′)** Example of photomicrographs of the brain regions analyzed for each experimental group: virgin/buttons, virgin/pups, late-pregnant/buttons, and late-pregnant/pups. Images correspond to the AOB **(A′)**, the PMCo **(B′)**, the MePD **(C′)**, and the BSTMPM **(D′)**. Scale bars, 250 μm **(A–D)** and 100 μm **(A′-D′)**. **(A″-D″)** Bar histogram showing the cFos positive cell density (mean ± SEM) in the vomeronasal system. Individual data are also indicated, with empty circles corresponding to the females displaying pup-directed aggression. Raw data are represented although they were log transformed for statistical analysis when necessary (AOB, PMCo, MePD, see text). Significant main effects revealed by the statistical analysis are indicated for each histogram. When FEMALE × STIMULUS interaction is observed, the results of *post-hoc* pairwise comparisons are indicated using asterisks: ***p < 0.001, **p < 0.01, and *p < 0.05.

Then, we explored cFos expression in primary and secondary vomeronasal brain centers. For the AOB (Figure 3A), a two-way ANOVA of Log cFos-ir area fraction revealed a significant main effect for STIMULUS (*F*_1, 19_ = 4.527, *p* = 0.047), but no significant differences for FEMALE (*F*_1, 19_ = 0.272, *p* = 0.608) neither FEMALE × STIMULUS interaction (*F*_1, 19_ = 1.288, *p* = 0.270). Thus, pups evoked a higher expression of c-Fos in the AOB as compared to buttons in both groups of females (Figures 3A–A″).

However, for the secondary vomeronasal brain centers, e.g., the PMCo (vomeronasal cortex), the MePD and the BSTMPM, statistical analysis revealed further significant differences. Thus, the two-way ANOVA of Log cFos-ir cell density in the PMCo (Figure 3B) showed a significant main effect for STIMULUS (*F*_1, 26_ = 51.313, *p* < 0.001) and significant FEMALE × STIMULUS interaction (*F*_1, 26_ = 21.597, *p* < 0.001), but no differences were found for FEMALE factor (*F*_1, 26_ = 2.346, *p* = 0.138). *Post-hoc* pairwise comparisons revealed that pups elicited higher response in LP than in virgin females (*p* = 0.037), whereas buttons raised higher response in the virgin group than in the pregnant females (*p* < 0.001). In the LP group, pups elicited higher cFos response than buttons (*p* < 0.001), whereas this difference did not reach significance in virgin females (*p* = 0.077) (Figures 3B–B″). Likewise, the two-way ANOVA of Log cFos-ir cell density in the MePD showed a significant main effect of STIMULUS (*F*_1, 26_ = 81.312, *p* < 0.001) and FEMALE × STIMULUS interaction (*F*_1, 2_ = 15.27, *p* = 0.001), but no main effect of FEMALE (*F*_1, 26_ = 1.143, *p* = 0.295). *Post-hoc* analysis of these effects revealed that pups elicited higher cFos response than buttons in both LP (*p* < 0.001) and virgins (*p* = 0.001) (Figures 3C–C″). On the other hand, exposure to buttons elicited a higher level of cFos in virgins than LP (*p* = 0.002), but interfemale differences in pup-induced cFos-ir cell density did not reach significance (*p* = 0.055). Overall, our results revealed that pup exposure induced a higher neuronal response in the PMCo (not significant for the MePD) of LP vs. virgin females, whereas buttons, used as neutral vomeronasal stimulus, induced a lower neuronal response in LP than in virgin females in both brain areas.

Concerning the BSTMPM, Wilcoxon test comparing stimuli rendered significant differences (*Z* = −2.426, *p* = 0.015), with pups eliciting higher cFos levels than buttons, whereas comparison of females did not reveal significant differences (*Z* = −0.725, *p* = 0.469) (Figures 3D–D″). The pattern of activity in the BSTMPM (cFos expression) in the different females exposed to pups and buttons, was similar to the one observed for VNO and AOB, and different to the one seen in PMCo and MePD.

In sum, the pattern of cFos expression observed in response to pups and buttons differs between LP and virgin females in some secondary vomeronasal centers (PMCo and MePD), whereas both groups of females show similar response in the VNO, AOB, and BSTMPM. In general, pups elicit more activation than buttons, thus suggesting that buttons are a good control stimulus for vomeronasal stimulation.

### Response of the Main Olfactory System to Pup-Derived Stimuli

**Figure 4:**
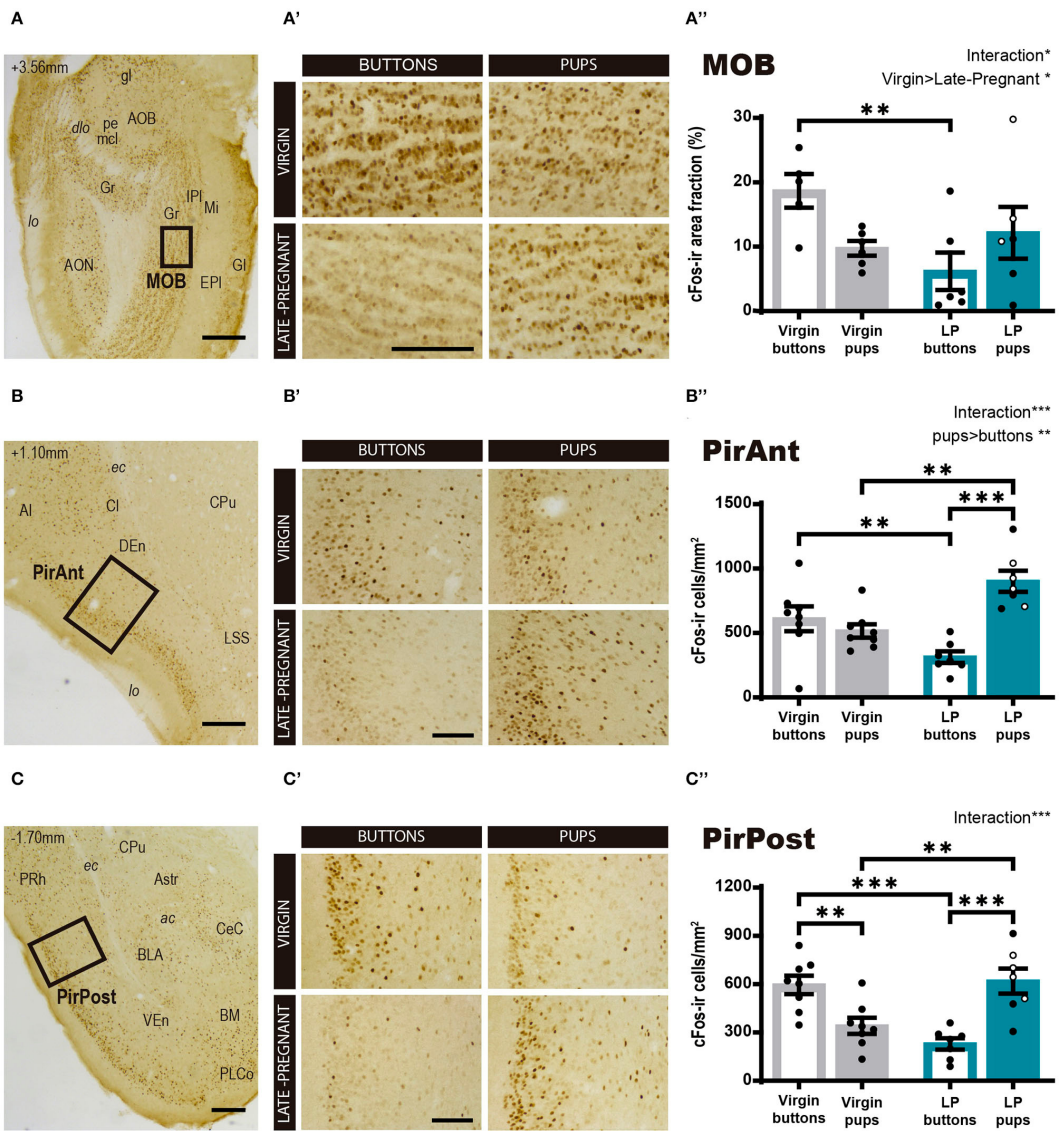
cFos response in brain areas of the main olfactory system following exposure to pups or non-social control stimulus. **(A–D)** Low power photomicrographs showing the cFos immunohistochemistry in the MOB **(A)**, the PirAnt **(B)**, and the PirPost **(C)**. The numbers in the left upper corner of the images indicate anteroposterior levels of the sections relative to bregma (Paxinos and Franklin, 2004). Framed areas, shown at higher magnification in **(A′-C′)**, indicate the specific zones where cFos expression was analyzed. **(A′-C′)** High-power photomicrographs of the brain centers analyzed for each experimental group: virgin/buttons, virgin/pups, late-pregnant/buttons, and late-pregnant/pups. Images correspond to the MOB **(A′)**, the PirAnt **(B′)**, and the PirPost **(C′)**. Scale bars, 250 μm **(A–C)** and 100 μm **(A′-C′)**. **(A″-C″)** Bar histogram showing the density (mean ± SEM) of cFos-expressing cells in the main olfactory system (raw data are displayed although data were log transformed for statistical analysis in the MOB, see text). Individual data are also indicated, with empty circles corresponding to the females displaying pup-directed aggression. Significant main effects revealed by the statistical analysis are indicated for each histogram. When FEMALE × STIMULUS interaction is observed, the results of *post-hoc* pairwise comparisons are indicated using asterisks: ***p < 0.001, **p < 0.01, and *p < 0.05.

Regarding the main olfactory system, we studied the cFos response in the MOB and the olfactory cortex, PirAnt and PirPost, following pups or neutral stimulus exposure in LP and virgin mice. The statistical analysis for Log cFos-ir area fraction in the MOB showed significant differences for FEMALE (*F*_1, 19_ = 5.611, *p* = 0.029) in favor of virgins, and a FEMALE × STIMULUS interaction effect (*F*_1, 19_ = 4.678, *p* = 0.044), whereas no differences were found for STIMULUS condition (*F*_1, 19_ = 0.027, *p* = 0.872). *Post-hoc* analysis of the interaction revealed that interfemale differences are mainly due to a significantly higher response of virgins to buttons as compared to LP females (*p* = 0.006) (Figures 4A–A″), as females do not differ in their response to pups. Those results evidence that pups do not evoke a different response between female groups, but buttons raised a MOB response higher in virgins than in LP mice.

On the other hand, the ANOVA for the cFos-ir cell density in the PirAnt showed significant differences for STIMULUS (*F*_1, 26_ = 11.425, *p* = 0.002; pups elicit more cFos-ir than buttons) and a FEMALE × STIMULUS interaction effect (*F*_1, 26_ = 21.748, *p* < 0.001), but no significant main effect of FEMALE factor (*F*_1, 26_ = 0.358, *p* = 0.555). *Post-hoc* comparisons showed that pups elicited higher cFos-ir density than buttons in LP females (*p* < 0.001) but not in virgins (Figures 4B–B″). In fact, when comparing animals exposed to pups, LP females displayed significantly higher cFos-ir than virgin animals (*p* = 0.001), and conversely, buttons elicited higher cFos expression in virgin than in LP females (*p* = 0.008).

For the PirPost a two-way ANOVA showed a significant FEMALE × STIMULUS interaction (*F*_1, 26_ = 32.209, *p* < 001), but no significant main effects for either FEMALE (*F*_1, 26_ = 0.620, *p* = 0.438) or STIMULUS (*F*_1, 26_ = 1.436, *p* = 0.242) (Figures 4C–C″). *Post-hoc* analysis showed that pups elicited higher response in LP than in virgin females (*p* = 0.002), while buttons induced higher response in virgins compared to LP females (*p* < 0.001). Moreover, differential effects of the stimuli were found within each female condition. Thus, LP females showed higher response to pups than to buttons (*p* < 0.001), whereas virgin females had a higher response to buttons than to pups (*p* = 0.003). Altogether, our results of neuronal activation in centers of the main olfactory system prompts to a differential stimulus discrimination, with higher activation by buttons in virgin females and by pup-derived stimuli in LP females.

### Correlation Analysis of Female Behavior and IEGs Expression

Finally, we performed correlation analysis between behavioral measures and the levels of IEGs expression in the vomeronasal and olfactory systems of virgin and LP female mice exposed to pups. Spearman analysis revealed a different pattern of correlation in LP and virgin females (see Table 1, Supplementary Figure 2). Thus, LP, but not virgins, displayed a significant positive correlation between “off nest” behavior and the cFos response in the AOB (*p* = 0.020) and the MOB (*p* = 0.001). On the contrary, the behavior “approach to pups,” consisting in sniffing a pup far from the nest without retrieving it afterwards, showed a significant positive correlation to cFos response in the AOB (*p* = 0.011) and the MePD (*p* = 0.017) in virgins, but not in LP females. Moreover, “nest building” was significantly and positively correlated with cFos response in the PirAnt (*p* = 0.011) in LP females, but not in virgins.

**Table 1:** Correlation analysis of IEGs expression in the VNO and centers of the chemosensory systems with behavior in late-pregnant (blue) and virgin females (light gray) exposed to pups.

			**NUCLEUS**							
**Behavior**	**Female group**	**Statistics**	**VNO**	**AOB**	**PMCo**	**MePD**	**BSTMPM**	**MOB**	**PirAnt**	**PirPost**
Off nest	Virgin	*r*s	0.454	0.617	0.172	0.061	−0.278	0.370	−0.098	0.049
		*p*-value	0.258	0.192	0.684	0.885	0.505	0.470	0.817	0.908
		*N*	8	6	8	8	8	6	8	8
	Late pregnant	*R*s	0.561	0.883^*^	0.112	0.393	0.559	0.971^**^	0.449	0.374
		*p-*value	0.190	**0.020**	0.811	0.383	0.249	**0.001**	0.312	0.408
		*N*	7	**6**	7	7	6	**6**	7	7
Approach to pups	Virgin	*r*s	−0.350	0.912^*^	0.350	0.801^*^	0.164	−0.441	0.626	0.175
		*p-*value	0.395	**0.011**	0.395	**0.017**	0.699	0.381	0.097	0.678
		*N*	8	**6**	8	**8**	8	6	8	8
	Late pregnant	*r*s	−0.482	−0.116	−0.556	0.148	0.030	−0.290	−0.148	−0.704
		*p-*value	0.274	0.827	0.195	0.751	0.954	0.577	0.751	0.077
		*N*	7	6	7	7	6	6	7	7
On nest	Virgin	*r*s	−0.024	−0.657	−0.476	−0.143	0.263	−0.086	−0.143	−0.238
		*p-*value	0.955	0.156	0.233	0.736	0.528	0.872	0.736	0.570
		*N*	8	6	8	8	8	6	8	8
	Late pregnant	*r*s	0.000	−0.152	−0.185	−0.630	−0.290	−0.395	0.185	0.037
		*p-*value	1.000	0.774	0.691	0.129	0.577	0.439	0.691	0.937
		*N*	7	6	7	7	6	6	7	7
Nest building	Virgin	*r*s	0.447	0.555	−0.243	0.089	0.373	−0.123	0.153	−0.128
		*p-*value	0.267	0.252	0.563	0.833	0.363	0.816	0.717	0.763
		*N*	8	6	8	8	8	6	8	8
	Late pregnant	*r*s	0.624	0.525	0.208	−0.567	0.359	0.309	0.869^*^	0.624
		*p-*value	0.135	0.285	0.655	0.184	0.485	0.552	**0.011**	0.135
		*N*	7	6	7	7	6	6	**7**	7
Pup retrieval	Virgin	*r*s	0.013	0.541	0.741^*^	0.294	−0.707	0.439	0.192	0.396
		*p-*value	0.976	0.268	**0.036**	0.480	0.050	0.383	0.650	0.332
		*N*	8	6	**8**	8	8	6	8	8
	Late pregnant	*r*s	−0.165	0.088	0.863^*^	−0.441	−0.206	0.000	−0.092	−0.165
		*p-*value	0.723	0.868	**0.012**	0.323	0.695	1.000	0.845	0.723
		*N*	7	6	**7**	7	6	6	7	7
In nest	Virgin	*r*s	−0.287	0.029	0.108	−0.252	0.108	−0.348	−0.168	−0.120
		*p-*value	0.490	0.957	0.799	0.548	0.798	0.499	0.691	0.778
		*N*	8	6	8	8	8	6	8	8
	Late pregnant	*R*s	−0.056	−0.324	0.150	0.636	0.093	0.000	−0.487	0.150
		*p-*value	0.905	0.531	0.749	0.125	0.862	1.000	0.268	0.749
		*N*	7	6	7	7	6	6	7	7
Maternal score	Virgin	*r*s	−0.096	−0.029	0.192	−0.240	−0.145	0.203	−0.144	0.048
		*p-*value	0.821	0.957	0.649	0.568	0.733	0.700	0.734	0.910
		*N*	8	6	8	8	8	6	8	8
	Late pregnant	*r*s	0.143	−0.086	0.821^*^	0.000	−0.029	0.200	−0.143	0.321
		*p-*value	0.760	0.872	**0.023**	1.000	0.957	0.704	0.760	0.482
		*N*	7	6	**7**	7	6	6	7	7
Chemosensory score	Virgin	*r*s	−0.405	0.086	0.238	−0.024	0.012	−0.143	0.000	−0.048
		*p-*value	0.320	0.872	0.570	0.955	0.978	0.787	1.000	0.911
		*N*	8	6	8	8	8	6	8	8
	Late pregnant	*r*s	−0.393	−0.371	−0.214	0.821^*^	−0.029	−0.086	−0.714	−0.357
		*p-*value	0.383	0.468	0.645	**0.023**	0.957	0.872	0.071	0.432
		*N*	7	6	7	**7**	6	6	7	7
Pup aggression score	Virgin	*r*s	–	–	–	–	–	–	–	–
		*p-*value	–	–	–	–	–	–	–	–
		*N*	8	6	8	8	8	6	8	8
	Late pregnant	*r*s	0.371	0.941^**^	−0.556	0.259	0.812^*^	0.698	0.259	0.408
		*p-*value	0.413	**0.005**	0.195	0.574	**0.050**	0.123	0.574	0.364
		*N*	7	**6**	7	7	**6**	6	7	7
Interfemale interaction	Virgin	*r*s	−0.291	0.120	0.048	0.436	0.122	0.000	0.509	0.097
		*p-*value	0.484	0.822	0.909	0.280	0.774	1.000	0.197	0.819
		*N*	8	6	8	8	8	6	8	8
	Late pregnant	*r*s	−0.374	−0.463	0.019	−0.412	−0.030	−0.772	−0.337	−0.056
		*p-*value	0.408	0.355	0.968	0.359	0.954	0.072	0.460	0.905
		*N*	7	6	7	7	6	6	7	7

* *p < 0.05*;

** *p < 0.01. Bold values indicate statistically significant correlation*.

The behavior “pup retrieval” was the only one displaying a significant positive correlation to neuronal response in the PMCo of both virgin females (*p* = 0.036) and in LP females (*p* = 0.012). In addition, in LP but not virgins, maternal score showed a significant positive correlation with the response in the PMCo (*p* = 0.023) and chemosensory score to the response in the MePD (*p* = 0.023). Finally, pup-directed aggression score correlated to the neural response in the AOB (*p* = 0.005) and in the BSTMPM (*p* < 0.05) only in LP females. The behaviors “on nest,” “in nest,” and “interfemale interaction” did not correlate with IEGs expression in any vomeronasal/olfactory brain region analyzed. In fact, Egr1 expression in the VNO did not correlate with any behavioral item or score. Overall, those positive correlations showed that in LP females, pup-directed and non-directed behaviors are mainly correlated to signal processing in some of the vomeronasal-related nuclei.

Finally, we also performed a correlation analysis of the levels of IEGs expression between vomeronasal and olfactory structures of females exposed to pups (Table 2, Supplementary Figure 3). Interestingly, this analysis revealed that VNO activation correlated with cFos expression in the MOB (*p* = 0.042), the PirAnt (*p* = 0.023), and the PirPost (*p* = 0.003) of LP females, while no similar correlations were found in virgins. Moreover, in LP females (but not virgins) AOB response displayed a positive correlation with MOB (*p* = 0.042) and with the BSTMPM (*p* < 0.001), whereas MOB and BSTMPM display no mutual correlation. By contrast, in virgin females, the BSTMPM displayed a significant negative correlation to PMCo (*p* = 0.040) and to MOB (*p* = 0.036), whereas the PirAnt positively correlated to MePD (*p* = 0.002) and the PirPost (*p* = 0.047).

**Table 2:** Correlation analysis of the IEGs expression between the different centers of the chemosensory systems (including the VNO) in late-pregnant (blue) and virgin females (light gray) exposed to pups.

		**VNO**	**AOB**	**PMCo**	**MePD**	**BSTMPM**	**MOB**	**PirAnt**	**PirPost**
VNO	rs		−0.257	−0.548	−0.524	0.072	0.714	−0.452	−0.119
	*p-*value		0.623	0.160	0.183	0.866	0.111	0.260	0.779
	*N*		6	8	8	8	6	8	8
AOB	*r*s	0.600		0.486	0.714	0.145	−0.314	0.771	0.143
	*p-*value	0.208		0.329	0.111	0.784	0.544	0.072	0.787
	*N*	6		6	6	6	6	6	6
PMCo	*r*s	0.071	−0.143		0.667	−0.731^*^	0.143	0.643	0.690
	*p-*value	0.879	0.787		0.071	**0.040**	0.787	0.086	0.058
	*N*	7	6		8	**8**	6	8	8
MePD	*r*s	0.071	0.200	−0.464		−0.240	−0.143	0.905^**^	0.571
	*p-*value	0.879	0.704	0.294		0.568	0.787	**0.002**	0.139
	*N*	7	6	7		8	6	**8**	8
BSTMPM	*r*s	0.257	1.000^**^	−0.543	0.314		−0.841^*^	−0.228	−0.707
	*p-*value	0.623		0.266	0.544		**0.036**	0.588	0.050
	*N*	6	**5**	6	6		**6**	8	8
MOB	*r*s	0.829^*^	0.829^*^	0.029	0.486	0.700		0.029	0.657
	*p-*value	**0.042**	**0.042**	0.957	0.329	0.188		0.957	0.156
	*N*	**6**	**6**	6	6	5		6	6
PirAnt	*r*s	0.821^*^	0.543	0.036	−0.321	0.314	0.543		0.714^*^
	*p-*value	**0.023**	0.266	0.939	0.482	0.544	0.266		**0.047**
	*N*	**7**	6	7	7	6	6		**8**
PirPost	*r*s	0.929^**^	0.543	0.143	0.036	0.143	0.771	0.679	
	*p-*value	**0.003**	0.266	0.760	0.939	0.787	0.072	0.094	
	*N*	**7**	6	7	7	6	6	7	

* *p < 0.05*;

** *p < 0.01. Bold values indicate statistically significant correlation*.

Overall, those results suggest that when exposed to pups LP females display an associated activity of the olfactory and vomeronasal systems. By contrast, in virgins, correlations mirror somehow the connectivity within the vomeronasal and olfactory pathways.

## Discussion

In the present study, we explored pregnancy-induced adaptations of the response of the chemosensory systems to pups in female mice. To do so, we analyzed the expression of IEGs in the VNO, as well as in the main centers of the vomeronasal and olfactory systems of virgin and LP female mice, in response to pups or to a non-social stimulus (buttons). This allows assessing changes in sensory processing of pup-derived chemosignals occurring by the end of pregnancy, most likely associated to the action of pregnancy hormones known to be relevant for inducing full maternal behavior. Last, we ascertained possible correlations between patterns of brain activity and behavior. Overall, our results confirm that pup-derived chemosignals activate the VNO and reveal changes in stimulus processing in chemosensory systems by the end of pregnancy. In addition, our data suggest that activation of different vomeronasal pathways are likely underlying pup care or pup attack in LP females.

### Methodological Issues and Behavioral Response to Pups

Our experimental design has several advantages. First, it prevents a differential novelty effect of pups in late-pregnant and virgin females, since both female groups were completely pup-naïve. This is relevant since novelty has a strong impact on exploratory behaviors (Rinaldi et al., 2010). Second, the use of a non-social control stimulus, buttons, is another advantage, since using non-exposed animals as controls does not allow to interpret IEGs expression as due to a specific stimulus (pups). Third, the females were housed in pairs at least 20 days before the experiment, and tested also in pairs, which avoided isolation stress along the procedure. Moreover, in order to avoid possible competition for stimuli between both females, we introduced a large number of pups/buttons into the test cage (eight), so that both females could interact with them simultaneously and independently. Fourth, when designing a IEGs experiment, using LP instead of postpartum females to check the activity induced by pups, has the additional advantage that does not require mother-infant separation. This suppresses another potentially confounding factor for interpreting the expression of IEGs in brain centers, e.g., pup-separation-induced stress (Aguggia et al., 2013).

Moreover, we are aware of some caveats in our procedure. The presence of two females in the same cage during the experiment may have interfered in the procedure, as an adult female is a source of chemosignals. Nonetheless, we minimized this possibility as we paired same-condition females for a long period and objects used as stimuli were introduced in large number to avoid competition, as above described. In any case, there were no significant differences in interfemale interactions between groups exposed to pups, and therefore, differences in IEGs expression are unlikely due to this factor, as supported also by the behavior-IEG expression correlation analysis.

When analyzing the behavior of virgin and LP females during exposure to pups, we observed no differences in any pup-directed or non-pup-directed behavior item, except for pup-directed aggression performed by some LP females (4 females out of 7) (see below). Our results on that issue agree with previous reports showing that virgin female mice having no previous experience with pups do not display pup aversion. Instead, pups constitute a highly attractive stimulus for pup-naïve females (Stolzenberg and Rissman, 2011; Alsina-Llanes et al., 2015; Martín-Sánchez et al., 2015). In those previous reports, authors demonstrated that maternal females (lactating dams or pup-sensitized virgins) display faster pup retrieval compared to pup-naïve virgins; however, in those experiments maternal females had previous pup-experience, while control virgins did not. Therefore, the lack of differences in most of the measured behavioral items between LP and virgin females in our experiment is likely due to pups being an equally novel stimulus for both kinds of females.

Although expression of IEG by neurons in vomeronasal and olfactory centers is mainly driven by detection of chemical stimuli, pups also emit distress vocalizations that are relevant in the context of maternal behaviors (Smotherman et al., 1974), together with olfactory cues with multisensory integration occurring at the level of the primary auditory cortex (Cohen et al., 2011). Whether, and to what extent, these stimuli might contribute to IEG expression in secondary olfactory and vomeronasal centers is not known. However, an analysis of the response of neurons in the primary auditory cortex to pup vocalizations (Marlin et al., 2015) revealed very faint response in pup-naïve virgins, as compared to pup-experienced virgins and dams. Since our females, both LP and virgins, had no previous experience with pups before the trial, we can safely assume that most, if not all the activity (IEG expression) observed in the centers of the vomeronasal and olfactory systems, is due to pup-derived chemosignals.

### Vomeronasal System Function and Behavioral Response to Pups

There is solid evidence indicating that VNO-detected chemosignals are likely crucial for some pup-directed responses, both parental and infanticide (Kimchi et al., 2007; Tachikawa et al., 2013; Nakahara et al., 2016; Isogai et al., 2018). In that respect, our results on Egr1 expression in the VNO demonstrate that pups are a source of vomeronasal stimuli for adult females (Figures 2E,F; Supplementary Figures 1, 2). Unlike other previous reports, we use a control, non-social novel stimulus, and compare the Egr1 expression in the VNO induced by buttons to that induced by pups. A role of VNO-detected stimuli in maternal behavior was proposed by Lepri et al. (1985), who reported delayed pup retrieval in lactating dams that had undergone removal of the VNO, as compared to sham-operated dams. Moreover, mice with impaired VNO function (null-trpc2 mice) display reduced maternal care (Kimchi et al., 2007), deficient nest maintenance and reduced nursing (Hasen and Gammie, 2011). Taken together, these data strongly suggest that pups emit chemosignals that are detected by the VNO of females and mediate adult female-pup interactions in the context of motivated maternal behavior.

However, our results indicate that pup exposure did not elicit differential Egr1 expression in the VNO of LP and virgin females (Figure 2 and Supplementary Figure 1), thus suggesting that hormone-induced changes in neurogenesis during pregnancy (Oboti et al., 2015) or hormone-induced changes in sensory transduction at the level of the VNO (Dey et al., 2015) might not be very relevant in the context of detection of pup chemosignals. By contrast, those changes may be relevant for the response of females to adult male chemosignals (e.g., major urinary proteins, Dey et al., 2015) perhaps in the context of nest defense (Martín-Sánchez et al., 2015), which is displayed by LP females (Mann and Svare, 1982). This lack of differences in VNO response to pups between females makes very unlikely that changes in pup-directed behaviors associated to pregnancy are due to altered sensitivity of the VNO. Instead, they should be attributed to altered sensory processing in the CNS during pregnancy. In this respect, our results also indicate that although pups induced an increase in cFos expression in the AOB (as compared to buttons) of females, this response was indistinguishable between virgin and LP females exposed to pups and a similar situation is found in the BSTMPM (compare Figures 3A″ and D″). This suggests that, like sensory transduction of pup chemosignals in the VNO, response to pups in the AOB-BSTMPM pathway is not under strong influence of pregnancy hormones.

Otherwise, in the vomeronasal cortex (PMCo) and the medial amygdaloid nucleus (MePD) the response to pups was different between LP and virgin females. Surprisingly, this is due in part to a higher activation of both nuclei by buttons in virgins, as compared to LP females. Since buttons are non-social objects, we assume that they do not directly activate vomeronasal neurons, so that the differential activation of these nuclei between button-exposed LP and virgins may be due to other afferents rather than direct vomeronasal inputs, as will be discussed later.

Importantly, in both secondary vomeronasal nuclei, LP females show increased activation by pups as compared to buttons, whereas virgins only show this pup-specific increase in cFos expression in the MePD, but not in the PMCo (where both stimuli elicit a similar activation). Consequently, in the PMCo, pup-induced cFos expression is significantly higher in LP than virgins. This indicates that sensory processing in the AOB-PMCo and AOB-MePD is modified during pregnancy. In the PMCo, this differential discrimination results in significant pup-button differences occurring only in LP females, suggesting that pregnancy modifies the functioning of specific vomeronasal pathways resulting in pup-specific activation of the vomeronasal cortex. This may be associated to reported changes in gene expression of key genes for endocrine signaling (e.g., receptor for prolactin) in afferents to the PMCo, such as the AOB and medial amygdala, during peripartum period in mice (Canavan et al., 2011). In addition, PMCo neurons display estrogen and progesterone receptors in rodents (Hagihara et al., 1992; Shughrue et al., 1997; Mitra et al., 2003). Thus, the important changes in steroid hormone levels occurring during late pregnancy (progesterone withdrawal, estrogen rise) may affect neural processing in the PMCo, altering the response to pup chemosignals.

An interesting finding of this work is the highly significant correlation observed between pup retrieval and cFos activity in the PMCo in both, LP and virgins. This points to a previous unknown role of this neural structure in the control of maternal behavior (the vomeronasal cortex, Gutiérrez-Castellanos et al., 2014), which fits the impact of VNO lesion in pup retrieval (Lepri et al., 1985). In addition, in LP females (but not virgins), the expression of cFos in the PMCo shows a remarkable positive correlation with the maternal score, a weighted sum of episodes in which female's behavior reflects a maternal state (pup retrieval, nest building, in nest and on nest). Although this suggests a relationship between both phenomena, PMCo activity and maternal behavior, the causal relationship it is not clear, e.g., whether the PMCo becomes activated by pups' stimuli during LP female interaction with them, or the PMCo activation is part of the neural mechanism responsible of the induction of maternal behavior. The PMCo projects to the BMA and to some extent to the BLA (Gutiérrez-Castellanos et al., 2014) and these nuclei of the basolateral amygdala are involved in goal-directed (e.g., pup-directed) behaviors via its projections to the accumbens-ventral pallidum (Numan and Woodside, 2010). Therefore, it is tempting to suggest that the PMCo may influence motivational aspects of maternal behavior using intra-amygdaloid pathways.

Concerning the MePD, its pattern of activation during exposure to pups/buttons looks rather similar to the one found in the PMCo, with some slight significant differences, as previously described. The MePD shows a very strong expression of steroid hormone receptors (Hagihara et al., 1992; Shughrue et al., 1997; Mitra et al., 2003), and, compared to virgins, pregnant females display a significant increase in pSTAT5-immunoreactive cell density, probably induced by placental lactogens (Salais-López et al., 2017). Thus, the influence of pregnancy hormones in LP females may underlie the correlation of cFos expression in the MePD with the chemosensory score, a weighted average of the episodes in which female-infant interactions are likely to include chemoinvestigation of pups. In contrast, in virgins, cFos expression in the MePD significantly correlates with the number of episodes in which the female exhibits “pup approach,” a kind of risk-assessment behavior directed to pups, in which the female approaches a pup but retreats afterwards without trying to retrieve it. Although indirect, these data suggest that activity in the MePD is mainly related to chemosensory stimulation in both kinds of females, but in LP this mainly occurs in the context of maternal approaches to pups, whereas in virgins it seems more related to pup-directed exploratory behavior.

### Vomeronasal Function and Pup-Directed Aggression

The role of vomeronasal stimuli in pup-directed aggression has been well-established in males. Thus, Tachikawa et al. (2013) demonstrated that infanticide in sexually naïve male mice is VNO-dependent, and accordingly, virgin infanticide males displayed much higher pup-induced activation (evaluated as cFos expression) in the VNO and AOB than sexually experienced, paternal males (Tachikawa et al., 2013). In line with this, Nakahara et al. (2016) showed that pups induced activation of an atypical subpopulation of neurons in the VNO that expresses a specific gene in the OR family, Olfr692. More recently, Isogai et al. (2018) demonstrated the implication of V2R-expressing VNO cells in the detection of specific molecules covering pup's bodies during postpartum (salivary secretions from the dam; hemoglobin) that induce pup killing in virgin males. In contrast, Trouillet et al. (2019) suggested the involvement of V1R/Galphai2-detected volatiles in virgin male infanticide.

Although the number of LP females exhibiting and not exhibiting pup-directed aggression is not large enough to establish two groups and compare their brain activity using robust statistics tools, the correlations between occurrence of pup aggression and brain activity renders interesting results. Our data show a positive correlation of AOB and BSTMPM activation (cFos expression) with pup-aggression score in LP females (Table 1; Supplementary Figure 2). This suggests that some vomeronasal-detected pup chemosignals might induce attacks in LP females, as it occurs in males, although the kind of VNO receptors involved is still unknown. In males transition from infanticide (sexually naïve males) to paternal care (sexually experienced males) seems associated to altered sensitivity of Olfr692-expressing VNO cells to pups (Nakahara et al., 2016) probably due to changes in sensory transduction in Galphai2/V1R-expressing cells (Trouillet et al., 2019). By contrast, our results suggest that in females other mechanisms seem to be at play. Thus, according to our results, LP and virgin females show similar Egr1-ir cell density in the VNO in response to pups, differences being observed only in some central vomeronasal centers, such as the PMCo. This again suggests that pregnancy-induced altered functioning of central circuits, rather than changes in vomeronasal sensory transduction, might mediate infant-directed aggression observed in some LP females.

A highly significant positive correlation was also observed in the group of LP females, between pup-induced cFos expression in the two nuclei whose activity is correlated with pup-aggression: BSTMPM and AOB (Table 2, Supplementary Figure 3). Therefore, our data suggest that the AOB-BSTMPM pathway may be involved in pup-directed aggression. The effects of pregnancy hormones in the BSTMPM or its afferents (Salais-López et al., 2017) might promote a pattern of activity that would facilitate pup-attack in LP females.

The medial posterior BST, as part of the medial extended amygdala, is a heterogeneous brain region (Dong and Swanson, 2006a,b) involved in social behavior (part of the sociosexual brain network) including parenting, mating and aggressive behavior (Tsuneoka et al., 2015; Fukui et al., 2019). Studies carried out in males suggest that caring of pups or attacking them results from the activity of a specific circuit within the BST-preoptic area (Tsuneoka et al., 2015), and also that the estrogen receptor signaling in the BST is likely contributing to infanticidal behavior (Fukui et al., 2019). In addition, this nucleus is involved in the regulation of inter-male aggression by VNO-detected male chemosignals (see also Trouillet et al., 2019). Our data suggests that in females, the BSTMPM may be part of an activated brain circuit associated to pup attack by the end of pregnancy, but future studies are needed to explore this possibility.

By contrast, virgin females did not show pup attacks, and this might be related to the significant negative correlation observed in virgins (but not in LP females) between the activity in the PMCo/MOB and the one in the BSTMPM (see Table 2, Supplementary Figure 3). This suggests that tonic inhibition of the BSTMPM by these two nuclei inhibits pup attack in virgin females, whereas this inhibition may be reduced to a certain degree in some LP females, thus facilitating pup-directed aggression.

### Olfaction and Behavioral Response to Pups: Vomeronasal-Olfactory Integration

Pup chemosignals are also detected by the main olfactory epithelium and processed by the associated brain pathway to trigger maternal behaviors (Seegal and Denenberg, 1974; Belluscio et al., 1998; Wang and Storm, 2011; Fraser and Shah, 2014). At the level of the MOB, however, our results reveal no preferential activation by either stimulus, but a differential response of the two kinds of females, with virgins showing globally a higher cFos density than LP females. This is mainly due to buttons inducing significantly higher cFos activation in virgins than LP females and indicates that buttons are not olfactory neutral. Our results on the MOB also suggest that pups are a source of olfactory stimuli, with no changes in olfactory sensitivity to pups associated to pregnancy. By contrast, either pregnancy hormones reduce sensitivity to button-derived odorants (Kanageswaran et al., 2016), or more likely, LP females explore buttons to a lesser extent than virgins. Very likely, top-down centrifugal projections within the olfactory systems (de Olmos et al., 1978; Shipley and Ennis, 1996; Wachowiak, 2010; Mohedano-Moriano et al., 2012; Aqrabawi et al., 2016), change their activity during pregnancy resulting in a reduced chemoinvestigation of buttons, which constitute novel, salient stimuli for virgin females.

Concerning exploration of pups, even if pups induced similar levels of cFos expression in the olfactory bulbs of LP and virgin females, it is interesting to note that MOB and AOB cFos levels show a positive and very significantly correlation in LP but not in virgin females (Table 2, Supplementary Figure 3). In addition, cFos-expressing cell density in both olfactory bulbs correlate with the number of periods that LP females were off nest (Table 1, Supplementary Figure 2). These data suggest a coupled activation of both chemosensory systems during exploration of the cage, far from the nest, further reinforcing the view that the main and accessory olfactory pathways are not parallel systems, but they work in tandem and play complementary roles in chemical analysis of the environment (see Martinez-Garcia et al., 2009). Indeed, there is anatomical evidence indicating that the olfactory and vomeronasal pathways converge on several secondary centers (Cádiz-Moretti et al., 2013). Moreover, our data suggest that, at least during pregnancy, both chemosensory systems are functionally interrelated already in their first central rely, the main and accessory olfactory bulbs, as pointed out by Pardo-Bellver et al. (2017) using an electrophysiological approach. This olfactory-vomeronasal functional relationship also results in correlation of pup-induced Egr1 expression in the VNO with the MOB and the piriform cortex of LP females (see Table 2 and Supplementary Figure 3).

Although the anatomical substrate of the reciprocal influence between vomeronasal and olfactory systems (probably consistent of multiple indirect connections) is currently unknown, our findings indicate that functional coupling of MOB and AOB is probably associated to specific behaviors, such as chemoinvestigation of the environment (off nest, rather than pup-directed conducts), as reported by Pardo-Bellver et al. (2017). Our data also suggest that behavior-specific coupling is facilitated under some physiological circumstances, such as late pregnancy. A possible explanation for this could be an increased sniffing-induced vomeronasal pumping during investigation of the environment by LP females (Meredith and O'Connell, 1979), although more experiments are needed to test this hypothesis.

The pattern of cFos expression indicates that, in females, the activity of the olfactory cortex in response to the presence of pups or buttons is different to what we found in the main olfactory bulbs. Thus, whereas in LP the olfactory cortex is preferentially activated by pup odors, such higher activation does not occur in virgin females, which show a preferential activation by button odors instead, at least in the PirPost. Since the chemosensory score, likely related to detection of pup odors, does not differ between both kinds of females, these data suggest that processing of odorant stimuli through the olfactory pathway is altered during pregnancy favoring response to pup odors. This may reflect a role of the piriform cortex as an associative rather than a primary sensory cortex (see review by Haberly, 2001), where some neurons respond preferentially to rewarded odors (Schoenbaum and Eichenbaum, 1995; Meissner-Bernard et al., 2019). Functional changes induced in the brain of females by the action of pregnancy hormones might increase the rewarding properties of pup-derived stimuli (Londei et al., 1989) and, consequently, it would increase the response of Pir cells to this stimulus. Indeed, enhanced response to pups in the olfactory cortex of LP females might be caused by changes in gene expression observed during the peripartum in the Pir and other centers of the olfactory systems in mice, such as reduced expression of oxytocin receptor to less than a half or a 2- to 3-fold increase in the expression of prolactin receptor (Canavan et al., 2011).

Although the functional consequences of this pup-biased response of the piriform cortex in LP females are difficult to ascertain yet, it is tempting to suggest that pup-induced activity in the Pir may be related with the expression of both, pup-directed and to non-pup-directed maternal behaviors. In fact, in LP females (but not virgins) there is a positive, significant correlation between nest building episodes and cFos expression in the PirAnt (see Table 1, Supplementary Figure 2). This suggests that cFos-related activity in the olfactory cortex, at least in PirAnt, is not a mere consequence of pup chemoinvestigation, but probably has a causal role in the induction of maternal behaviors.

## Conclusions

In summary, our results reveal that pups are a source of chemical signals detected by the VNO, as demonstrated using quantitative assessment of Egr1 in the VNO. In LP females, processing of these chemosignals involves co-activation of the olfactory and vomeronasal systems, already at the level of the olfactory bulbs. Our data also depict two different subsystems within the vomeronasal system. On the one hand, in the pathway from the AOB to the PMCo and MePD sensory processing seems to be altered during late pregnancy so that discrimination between pups and buttons is enhanced. In addition, in LP females the activity in this pathway seems associated to pro-maternal behaviors, including pup retrieval and nest building. On the other hand, the pathway from the AOB to the BSTMPM shows no evidence of differential sensory processing in LP and virgin females. Although globally, activity in these centers is higher in pup- as compared to button-exposed females, within the group of LP females activity in both centers is correlated to pup-directed aggression, thus suggesting a role of vomeronasal stimulation of the BSTMPM in inducing pup attacks in females (during pregnancy), similar to what has been reported in virgin males for other portions of the BST.

## Data Availability Statement

The raw data supporting the conclusions of this article will be made available by the authors, without undue reservation.

## Ethics Statement

The animal study was reviewed and approved by Committee of Ethics and Animal Experimentation of the Universitat Jaume I.

## Author Contributions

CN-M, MB-M, and FM-G designed the experiments. CN-M, MS-C, MB-M, RG-C, and MB performed the experiments. CN-M, MS-C, and FM-G analyzed the data. CN-M, MS-C, EL, CA-P, and FM-G drafted the manuscript. All authors contributed to the article and approved the submitted version.

## Conflict of Interest

The authors declare that the research was conducted in the absence of any commercial or financial relationships that could be construed as a potential conflict of interest.

## Abbreviations

3V: 3rd ventricle
ac: anterior commissure
ADP: anterodorsal preoptic nucleus
AHiPM: amygdalohippocampal area, posteromedial part
AI: agranular insular cortex
AOB: anterior olfactory bulb
AON: anterior olfactory nucleus
APir: anterior lobe of pituitary
Astr: amygdalostriatal transition area
BLA: basolateral amygdaloid nucleus, anterior part
BLP: basolateral amygdaloid nucleus, posterior part
BM: basomedial amygdaloid nucleus
BMA: basomedial amygdaloid nucleus, anterior part
bl: basal layer
BSTIA: bed nucleus of the stria terminalis, intraamygdaloid division
BSTMPI: bed nucleus of the stria terminalis, posterointermediate part
BSTMPL: bed nucleus of the stria terminalis, posterolateral part
BSTMPM: bed nucleus of the stria terminalis, posteromedial part
CA3: field CA3 of the hippocampus
CeC: central amygdaloid nucleus, capsular part
CeL: central amygdaloid nucleus, lateral division
Cl: caudal interstitial nucleus of the medial longitudinal fasciculus
CPu: caudate putamen (striatum)
DAB: diaminobenzidine tetrahydrochloride
DEn: dorsal endopiriform nucleus
*dlo*: dorsal lateral olfactory tract
D3V: dorsal 3rd ventricle
EPI: external plexiform layer of the olfactory bulb
*ec*: external capsule
*f*: fornix
gl: glomerular layer of the olfactory bulb
Gr: granular layer of the olfactory bulbs
Gl: glomerular layer of the olfactory bulb
*ic*: internal capsule
IEGs: immediate early genes
IPl: inner plexiform layer of the olfactory bulbs
ir: immunoreactive
*lo*: lateral olfactory tract
LP: late-pregnant
LSS: lateral stripe of the striatum
MB: mushroom body of the VNO
mcl: mitral cell layer of the accessory olfactory bulb
mdl: midline
MePD: posterodorsal medial amygdala
MePV: medial amygdaloid nucleus, posteroventral part
Mi: mitral cell layer of the main olfactory bulb
MOB: main olfactory bulb
mv: microvillar layer
NSE: non-sensory epithelium of the VNO
OB: olfactory bulb
OE: olfactory epithelium
*opt*: optic tract
PB: phosphate buffer
PBS: phosphate saline buffer
pe: external plexiform layer of the accessory olfactory bulb
PFA: paraformaldehyde
PirAnt: anterior piriform cortex
PirPost: posterior piriform cortex
PLCo: posterolateral cortical amygdaloid nucleus
PMCo: posteromedial cortical amygdaloid nucleus
PRh: perirhinal cortex
SE: sensory epithelium of the VNO
*sm*: stria medullaris of the thalamus
*st*: stria terminalis
TB: tris buffer
TBS: tris saline buffer
Vd: vomeronasal duct
VEn: ventral endopiriform nucleus
Vn: vomeronasal nerve
VNO: vomeronasal organ
Vv: vomeronasal vein.
