# Supplementary material for: Pregnancy Changes the Response of the Vomeronasal and Olfactory Systems to Pups in Mice
Source: Front Cell Neurosci. 2020 Dec 18;14:593309. doi: 10.3389/fncel.2020.593309 (PMC7775479; doi:10.3389/fncel.2020.593309)
Supplement: Supplementary file 1 [file Presentation_1.PPTX]

## Slide 1
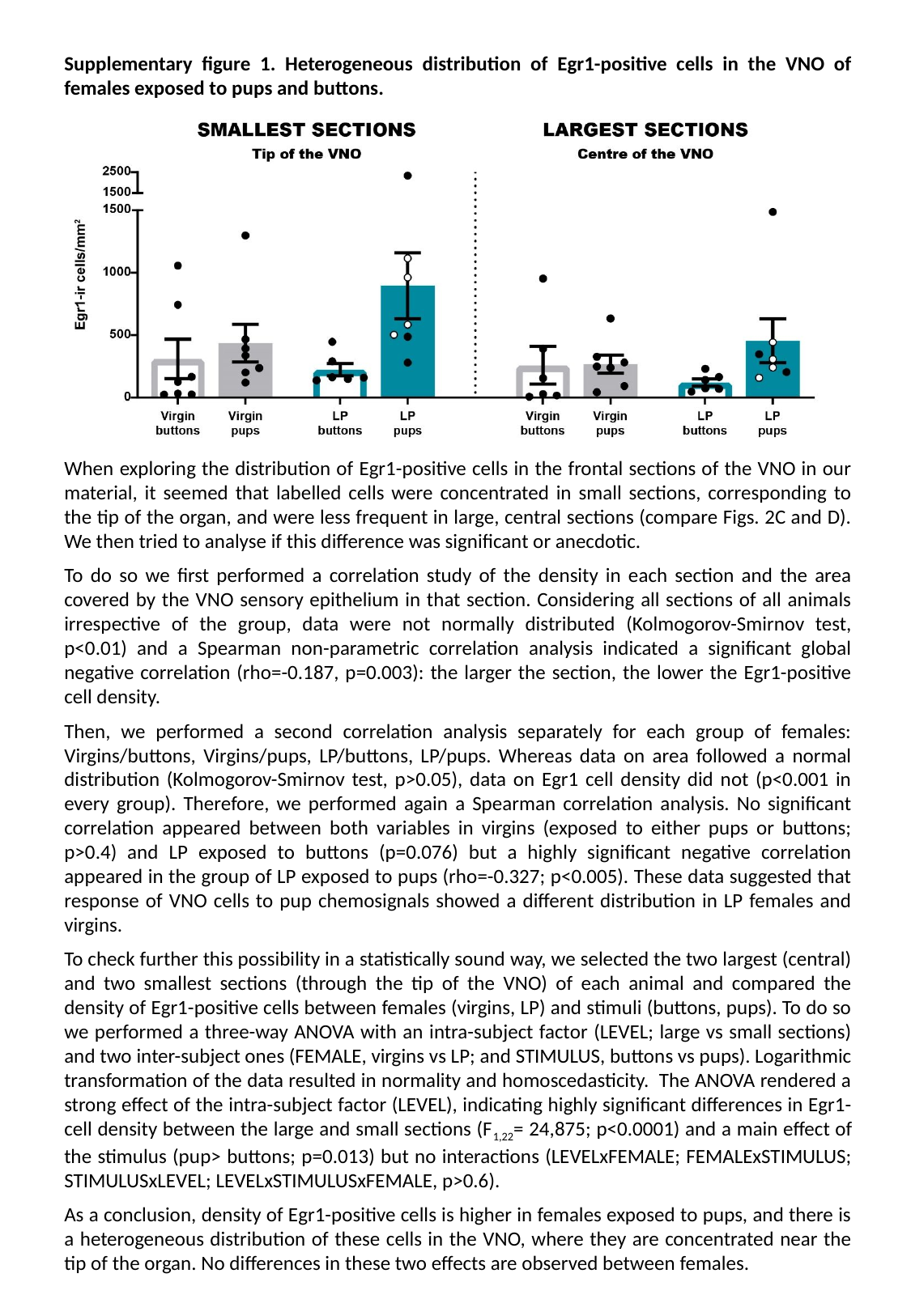

Supplementary figure 1. Heterogeneous distribution of Egr1-positive cells in the VNO of females exposed to pups and buttons.
When exploring the distribution of Egr1-positive cells in the frontal sections of the VNO in our material, it seemed that labelled cells were concentrated in small sections, corresponding to the tip of the organ, and were less frequent in large, central sections (compare Figs. 2C and D). We then tried to analyse if this difference was significant or anecdotic.
To do so we first performed a correlation study of the density in each section and the area covered by the VNO sensory epithelium in that section. Considering all sections of all animals irrespective of the group, data were not normally distributed (Kolmogorov-Smirnov test, p<0.01) and a Spearman non-parametric correlation analysis indicated a significant global negative correlation (rho=-0.187, p=0.003): the larger the section, the lower the Egr1-positive cell density.
Then, we performed a second correlation analysis separately for each group of females: Virgins/buttons, Virgins/pups, LP/buttons, LP/pups. Whereas data on area followed a normal distribution (Kolmogorov-Smirnov test, p>0.05), data on Egr1 cell density did not (p<0.001 in every group). Therefore, we performed again a Spearman correlation analysis. No significant correlation appeared between both variables in virgins (exposed to either pups or buttons; p>0.4) and LP exposed to buttons (p=0.076) but a highly significant negative correlation appeared in the group of LP exposed to pups (rho=-0.327; p<0.005). These data suggested that response of VNO cells to pup chemosignals showed a different distribution in LP females and virgins.
To check further this possibility in a statistically sound way, we selected the two largest (central) and two smallest sections (through the tip of the VNO) of each animal and compared the density of Egr1-positive cells between females (virgins, LP) and stimuli (buttons, pups). To do so we performed a three-way ANOVA with an intra-subject factor (LEVEL; large vs small sections) and two inter-subject ones (FEMALE, virgins vs LP; and STIMULUS, buttons vs pups). Logarithmic transformation of the data resulted in normality and homoscedasticity. The ANOVA rendered a strong effect of the intra-subject factor (LEVEL), indicating highly significant differences in Egr1-cell density between the large and small sections (F1,22= 24,875; p<0.0001) and a main effect of the stimulus (pup> buttons; p=0.013) but no interactions (LEVELxFEMALE; FEMALExSTIMULUS; STIMULUSxLEVEL; LEVELxSTIMULUSxFEMALE, p>0.6).
As a conclusion, density of Egr1-positive cells is higher in females exposed to pups, and there is a heterogeneous distribution of these cells in the VNO, where they are concentrated near the tip of the organ. No differences in these two effects are observed between females.

## Slide 2
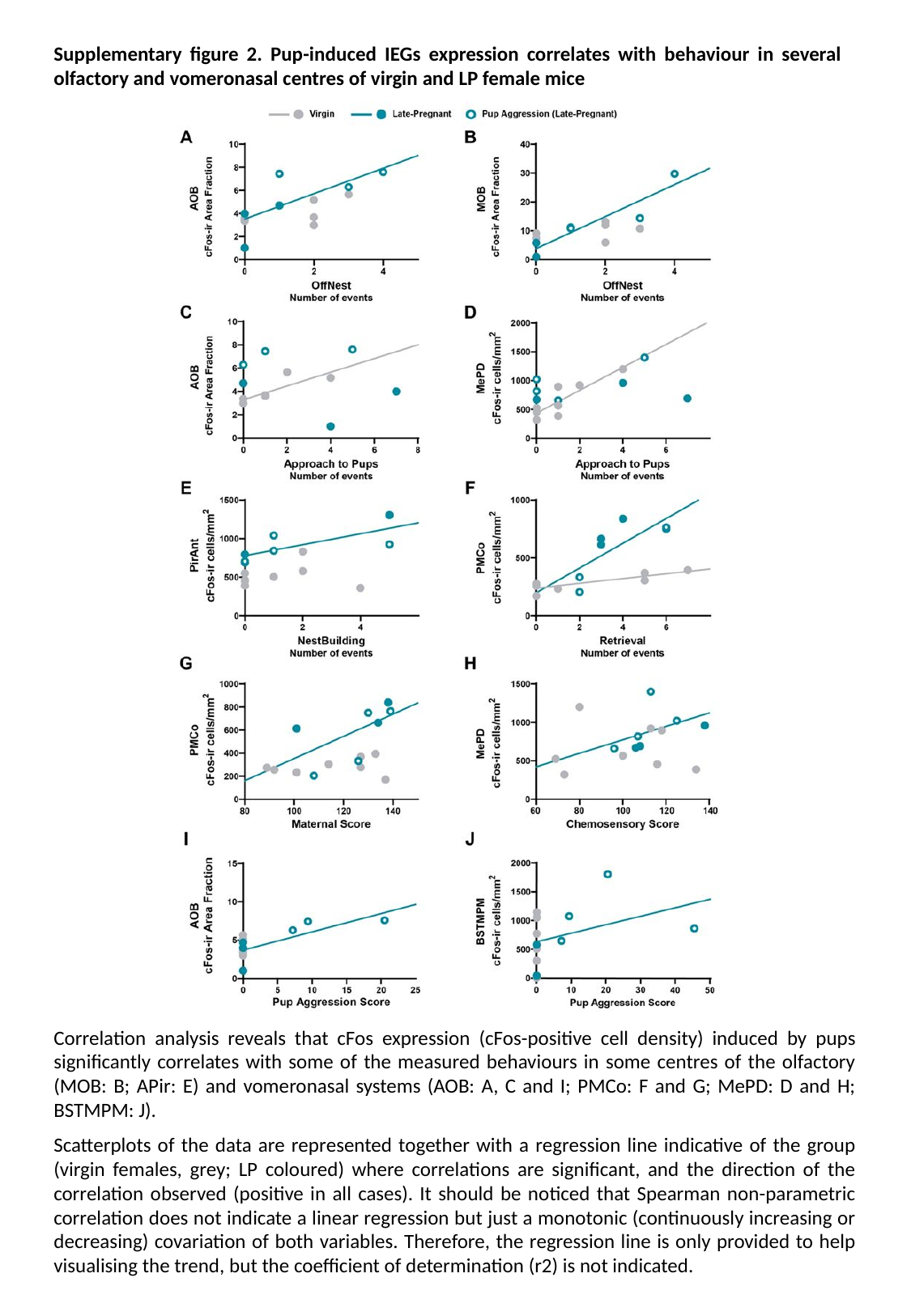

Supplementary figure 2. Pup-induced IEGs expression correlates with behaviour in several olfactory and vomeronasal centres of virgin and LP female mice
Correlation analysis reveals that cFos expression (cFos-positive cell density) induced by pups significantly correlates with some of the measured behaviours in some centres of the olfactory (MOB: B; APir: E) and vomeronasal systems (AOB: A, C and I; PMCo: F and G; MePD: D and H; BSTMPM: J).
Scatterplots of the data are represented together with a regression line indicative of the group (virgin females, grey; LP coloured) where correlations are significant, and the direction of the correlation observed (positive in all cases). It should be noticed that Spearman non-parametric correlation does not indicate a linear regression but just a monotonic (continuously increasing or decreasing) covariation of both variables. Therefore, the regression line is only provided to help visualising the trend, but the coefficient of determination (r2) is not indicated.

## Slide 3
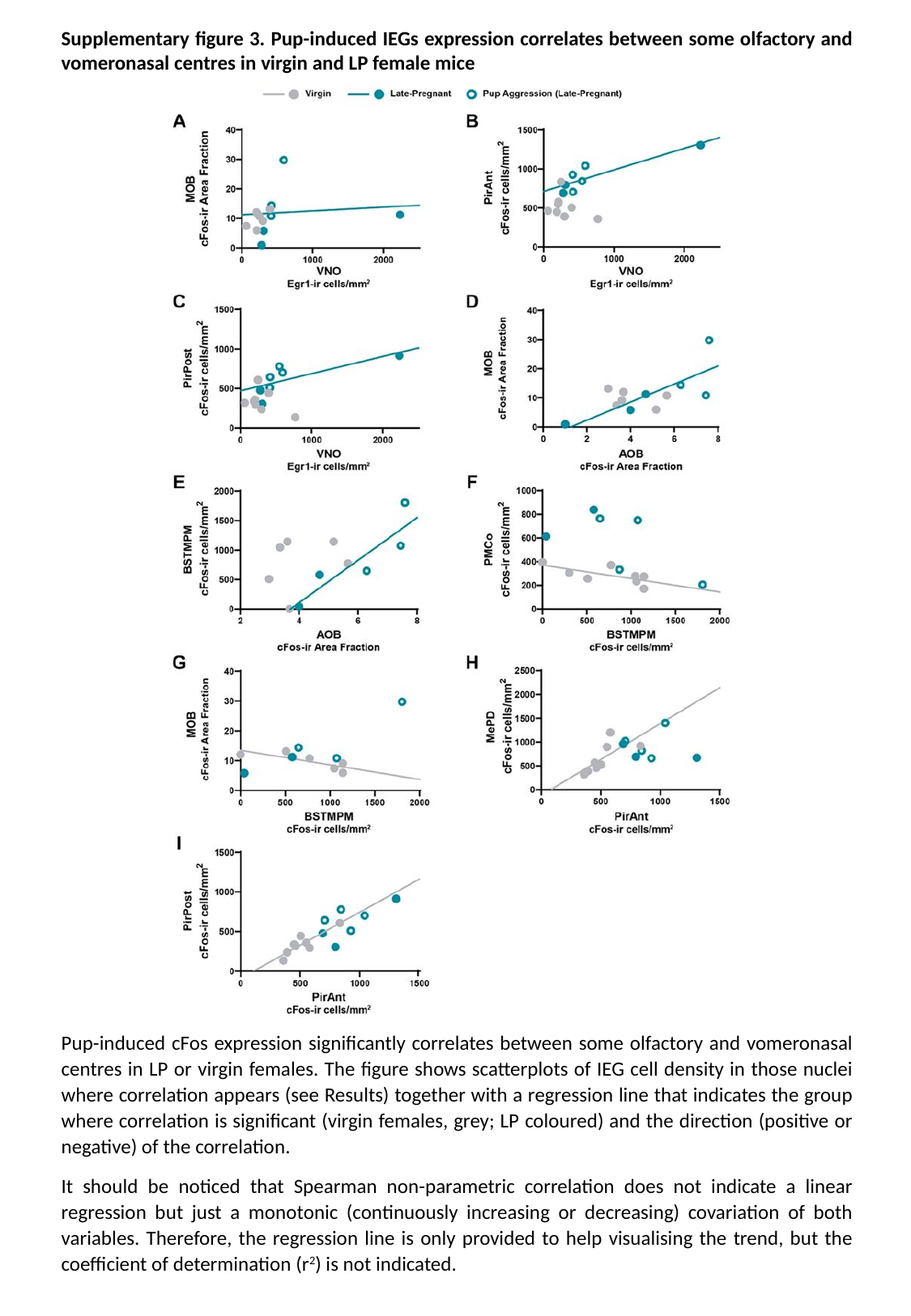

Supplementary figure 3. Pup-induced IEGs expression correlates between some olfactory and vomeronasal centres in virgin and LP female mice
Pup-induced cFos expression significantly correlates between some olfactory and vomeronasal centres in LP or virgin females. The figure shows scatterplots of IEG cell density in those nuclei where correlation appears (see Results) together with a regression line that indicates the group where correlation is significant (virgin females, grey; LP coloured) and the direction (positive or negative) of the correlation.
It should be noticed that Spearman non-parametric correlation does not indicate a linear regression but just a monotonic (continuously increasing or decreasing) covariation of both variables. Therefore, the regression line is only provided to help visualising the trend, but the coefficient of determination (r2) is not indicated.
